# Narrowing abdominal aorta and vein is a simple and useful method for preparing a mice model of gestational hypertension

**DOI:** 10.1016/j.heliyon.2019.e01576

**Published:** 2019-04-30

**Authors:** Yuri Yasui, Keiichi Kumasawa, Kenji Minato, Hitomi Nakamura, Tadashi Kimura

**Affiliations:** Department of Obstetrics and Gynecology, Graduate School of Medicine, Osaka University, 2-2 Yamadaoka, Suita, Osaka 565-0871, Japan

**Keywords:** Physiology

## Abstract

Hypertensive disorder of pregnancy (HDP) is a major cause of maternal morbidity and mortality, fetal growth restriction (FGR), and premature delivery. Soluble fms-like tyrosine kinase-1 (sFLT-1) is significantly elevated in pre-eclamptic women. Making animal models of hypertensive pregnancy is costly and requires advanced equipment. We established a gestational hypertension (GH), one of the HDP subtypes, mouse model by narrowing the abdominal aorta and vein together with a medical drip tube on day 10.5 of gestation. Systolic and diastolic blood pressure on day 18.5 of gestation in the narrowed aorta and vein (NAV) group were significantly higher than those in the control group. Fetal weight decreased in the NAV group. Serum sFLT-1 was significantly increased in the NAV group on day 18.5 of gestation compared to the control group. After delivery, blood pressure and serum sFLT-1 level did not differ between the NAV and the control groups. These parameters normalized postpartum. We established a novel GH mouse model through an easy operative procedure using a simple device. In this NAV model, blood pressure and serum sFLT-1 level were increased on day 18.5 of gestation, and normalized promptly after delivery. The mouse model mimics human GH, and is suitable for the development of other treatments.

## 1 Introduction

According to National High Blood Pressure Education Program (NHBPEP), hypertensive disorder of pregnancy (HDP) is classified as gestational hypertension, preeclampsia-eclampsia, superimposed preeclampsia on chronic hypertension and chronic proteinuria. HDP occurs in 2–7% of pregnant women [1]. It is a major cause of maternal morbidity and mortality, fetal growth restriction (FGR), and premature delivery. GH is diagnosed by systolic blood pressure of 140 mmHg or diastolic blood pressure of 90 mmHg for the first time after 20 weeks of gestation and without proteinuria or other end-organ dysfunction. GH is transient hypertension during pregnancy and the blood pressure returns to normal by 12 weeks postpartum. Almost half of GH women subsequently develop PE.

Currently soluble fms-like tyrosine kinase-1 (sFLT-1) has attracted much of the attention as an anti-angiogenic factor produced mainly by the placenta. Soluble FLT-1 is significantly elevated in women with PE or GH [2]. Furthermore, it begins to increase before the onset of PE or GH, therefore, a higher level of serum sFLT-1 is used to predict PE or GH [3, 4, 5, 6] and it is estimated that some of women with GH will develop preeclampsia [2, 7].

Animal models are very useful for providing explanations and developing treatment. Various animal models of PE or GH have been established. These are used frequently for studying anti-angiogenic factors [8, 9] and oxidative stress (reduced uterine perfusion pressure model) [10, 11, 12]. Recently, we explored the lentiviral vectors and expressed human sFLT-1 specifically in the murine placenta, and we reported the effectiveness of pravastatin for treatment [13]. However, constructing this animal model required advanced equipment and large amount of cost. Therefore, through an easy operation with the simple device we tried to establish a GH or PE mouse model.

## 2 Material and methods

### 2.1 Animal experiments

All experiments were approved by the Institutional Animal Care and Use Committee of Osaka University. Studies were performed in ICR mice obtained from Japan SLC, Inc. (Shizuoka, Japan). The mice were housed in the Institute of Experimental Animal Sciences Faculty of Medicine, Osaka University. Seven to 12-week-old female mice were mated with ICR males in the night. We checked the sperm plugs in the females the next morning. Moreover, we recognized this period as gestational day 0.5. The operation was performed on day 10.5 of gestation.

The operation was performed under anesthesia using three types of mixed anesthetic agents (midazolam, medetomidine, and butorphanol tartrate). We narrowed the aorta by using a medical drip tube of 23G-route. We cut the tube to a width of 2.3 mm and it was cut to unroll for setting. After a horizontal incision, the retroperitoneum was opened, and the abdominal aorta and vein were lifted using curved tweezers; then, the tube was placed in both the aorta and vein. We narrowed both abdominal aorta and vein together (NAV) with the facile device. We gently knotted the tube to prevent it from unrolling. On the other hand, the control group underwent abdominal section under the same anesthesia, the retroperitoneum was opened, and the abdominal aorta and vein were lifted using curved tweezers.

### 2.2 Measurement of blood pressure

Blood pressure was measured by the tail-cuff method with BP98A (Softron). We measured blood pressure on day 10.5 and 18.5 of gestation. The mice were gently placed in a small soft cage without being anesthetized. After stabilization of their behavior and heart rates, both systolic and diastolic blood pressures were recorded, the mean of 10 values was used for further statistical analysis.

### 2.3 Conceptus measurements

On day 18.5 of gestation, after measurement of blood pressure and body weight, we collected blood samples by cardiac puncture and urinary samples by bladder paracentesis under three types of mixed anesthetic agents, and the uterus was exteriorized. The number of fetuses and aborted fetuses were counted, and we measured the fetal and placental weight.

### 2.4 Measurement of plasma sFLT-1, placental growth factor (PlGF), VEGF

Blood samples were allowed to clot and were centrifuged to separate the blood from the serum samples. Concentration of serum mouse sFLT-1, mouse PlGF, mouse VEGF were measured with ELISA kits from R&D systems.

### 2.5 Measurement of urinary albumin and creatinine

Urine albumin and creatinine were measured by Fuji DRI−CHEM 3500V and DRI-CHEM slides (Fujifilm).

### 2.6 Statistical analysis and calculations

Data were presented as means ±SD. We conducted a comparison between groups by using the Student's *t*-Test. A *P* value of less than 0.05 was considered statistically significant.

## 3 Results

### 3.1 Influence of narrowing both abdominal aorta and vein together in a pregnant mouse on spontaneous abortion and litter size

**Fig. 1:**
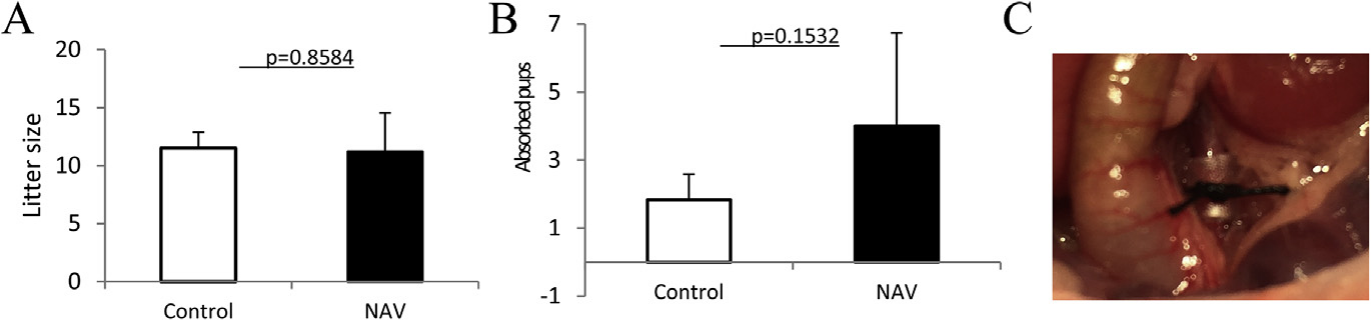
Influence of tubing both the abdominal aorta and vein together in a pregnant mouse on litter size and spontaneous abortion (A) Litter size was not significantly different between the NAV and the control groups on day 18.5 of gestation. (B) The number of reabsorbed pups on day 18.5 of gestation was slightly increased. The bar graph represents means ±SD. n = 6 for control and n = 5 for NAV. (C)We narrowed the aorta and vein by using a medical drip tube.

Litter size was not significantly different between the NAV and the control groups on day 18.5 of gestation (11.2 ± 3.3466 pups vs. 11.5 ± 1.3784 pups, *p* = 0.8584, Fig. 1A). The number of absorbed pups on day 18.5 of gestation was slightly increased (4.0 ± 2.7386 pups vs. 1.8 ± 0.7528 pups, *p* = 0.1532, Fig. 1B). Applying the tube to the abdominal aorta and vein did not induce abortion and did not influence the number of pups.

### 3.2 Blood pressure and urine albumin/creatinine ratio

**Fig. 2:**
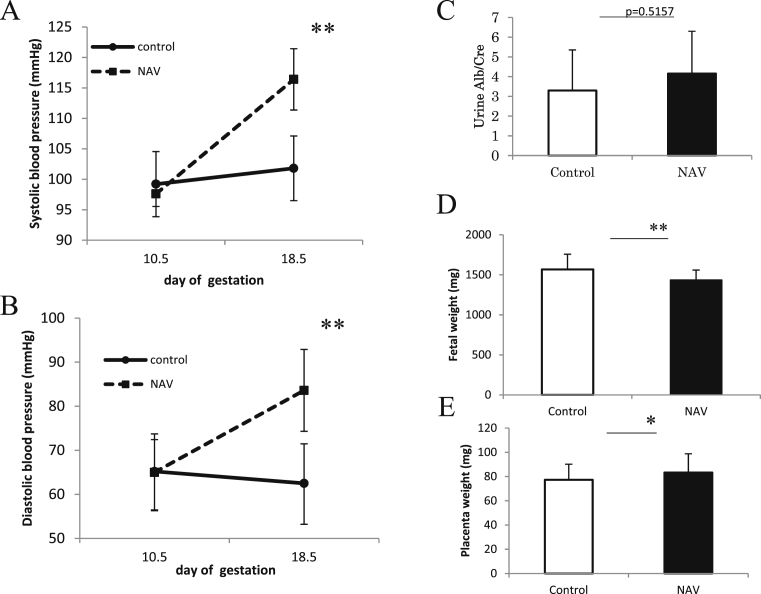
Influence of NAV on systolic blood pressure (A), diastolic blood pressure (B), urine Alb/Cre ratio (C), fetal weight (D) and placenta weight (E). Systolic and diastolic blood pressure on day 18.5 of gestation in the NAV group were significantly higher than those in the control group. Urine Alb/Cre ratio was not significantly different between the NAV and the control groups. Fetal weight decreased and placenta weight increased in the NAV group. The bar graph represents means ±SD. *p < 0.05, **p < 0.01 using unpaired Student's t-test (A), (B) (C) n = 6 for control and n = 5 for NAV. (D), (E) n = 69 for control and n = 56 for NAV.

Systolic and diastolic Blood pressure were measured on day 10.5 of gestation (before operation) and on day 18.5 of gestation. Systolic and diastolic blood pressure on day 18.5 of gestation in the NAV group were significantly higher than those in the control group (116.4 ± 5.03 mmHg vs. 101.8 ± 5.307 mmHg, *p* = 0.0012, Fig. 2A) (83.6 ± 9.29 mmHg vs. 62.5 ± 8.96 mmHg, *p* = 0.0041, Fig. 2B). Urine albumin/creatinine ratio was slightly increased but not significantly different compared to the control group (4.2 ± 2.145 vs. 3.3 ± 2.06, p = 0.5157, Fig. 2C). The results indicate that narrowing both the abdominal aorta and the vein induced hypertension during pregnancy but did not result in proteinuria.

### 3.3 Fetal weight and placenta weight

In the NAV group, fetal weight decreased compared to that of the control group (1436.1 ± 124.0 mg vs. 1567.6 ± 190.0 mg, p < 0.01, Fig. 2D). Placenta weight slightly increased in the NAV group (83.3 ± 15.5 mg vs. 77.3 ± 12.8 mg, 0.01 < p = 0.020 < 0.05, Fig. 2E).

### 3.4 Serum angiogenic factors

**Fig. 3:**
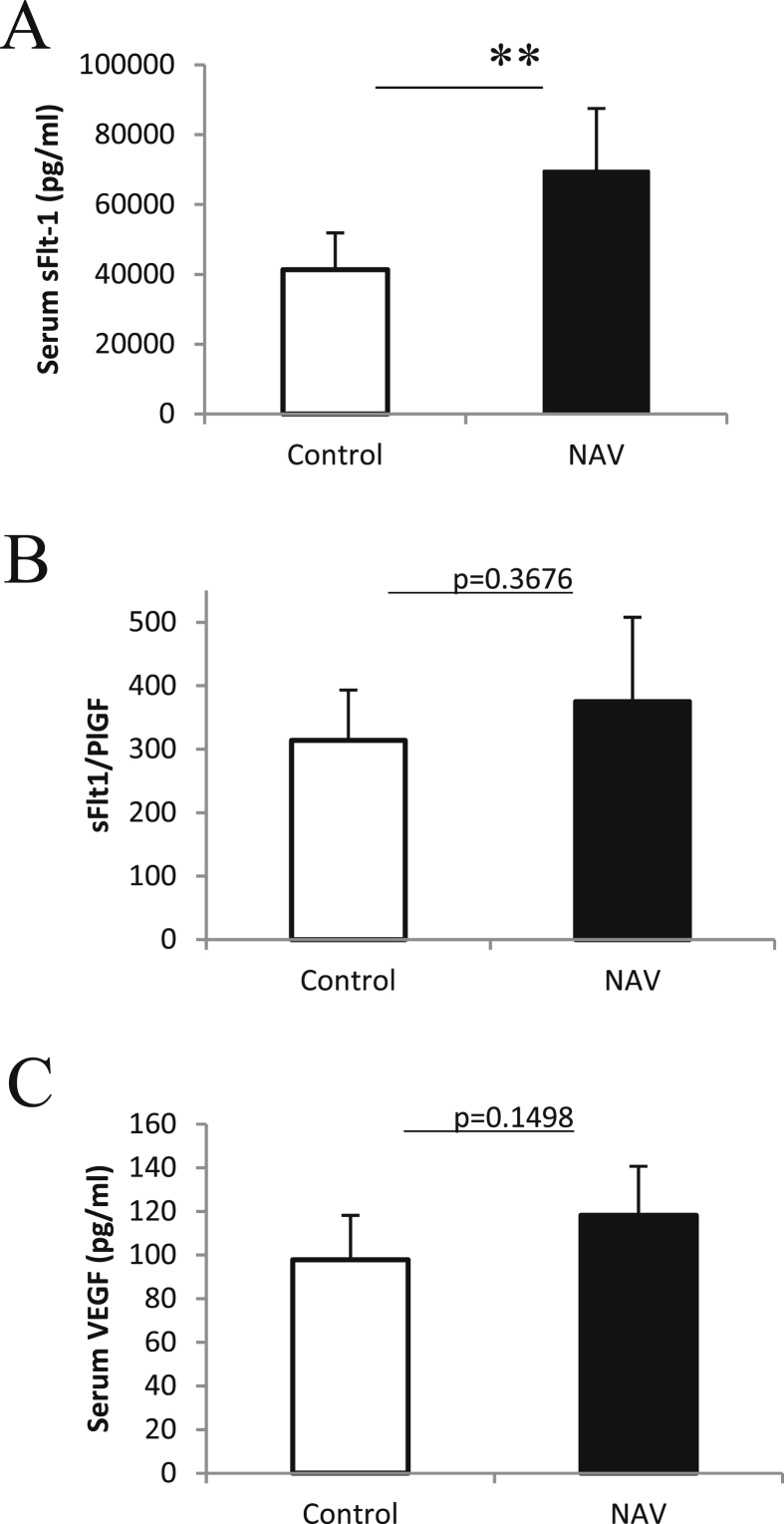
Serum angiogenic Factors by ELISA (A) Serum sFLT-1 level was significantly increased in the NAV group on day 18.5 of gestation compared to the control group. (B) (C) Serum sFLT-1/PlGF ratio and serum VEGF level was slightly increased in the NAV group. The bar graph represents means ±SD. **p < 0.01 using unpaired Student's t-test. n = 6 for control and n = 5 for NAV.

Serum sFLT-1 level was significantly increased in the NAV group on day 18.5 of gestation compared to the control group (69540.8 ± 17975.54 pg/ml vs. 41346.8 ± 10517.37 pg/ml, P < 0.01, Fig. 3A). Serum VEGF levels were slightly increased in the operation group but not differ significantly between the NAV and the control groups (118.18 ± 22.51 vs. 97.85 ± 20.34 pg/ml, p = 0.1498, Fig. 3B). Serum sFLT-1/PlGF ratio was slightly increased in the NAV group (375.03 ± 132.86 pg/ml vs. 313.84 ± 79.42 pg/ml, p = 0.3676, Fig. 3C).

### 3.5 Blood pressure and serum sFLT-1 level after delivery

**Fig. 4:**
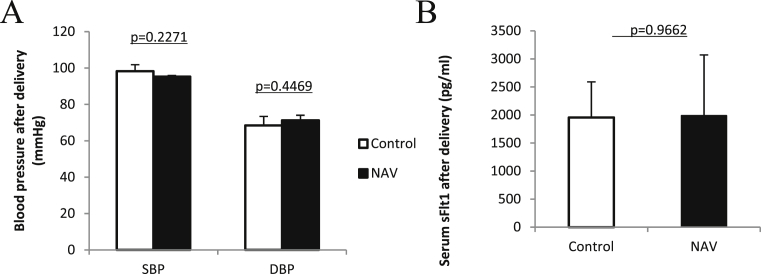
Blood pressure and serum sFLT-1 level after delivery (A) (B) Blood pressure and serum sFLT-1 level were not different between the NAV and the control groups. These were normalized promptly after delivery. The bar graph represents means ±SD. n = 3 for control and n = 3 for NAV.

The blood pressure was measured postpartum from 7 to 10 days. Blood pressures were not significantly different between the NAV and the control groups (95.27 ± 0.667 mmHg vs. 98.27 ± 3.58 mmHg, p = 0.2271) (71.23 ± 2.83 mmHg vs. 68.47 ± 4.93 mmHg, p = 0.4469) (Fig. 4A). Serum sFLT-1 level was almost the same in both groups (1988.19 ± 1082.75 pg/ml vs. 1955.54 ± 634.93 pg/ml, p = 0.9662, Fig. 4B). The blood pressure in the NAV group normalized postpartum, which mimics human recovery after delivery.

## 4 Discussion

We established a novel GH, one of the HDP subtypes, mouse model through easy operative techniques with the simple device. In the NAV model, serum sFLT-1 level and the blood pressure increased by narrowing the abdominal aorta and vein using a device but were normalized promptly after delivery. In addition, fetal growth restriction was accompanied with NAV group. Considering these, the NAV mouse model imitated human GH physiology.

Almost half of GH women subsequently develop PE. The mechanisms of PE have not yet been thoroughly elucidated. The ‘two-step theory’ is widely accepted [14, 15]. In this theory, poorly perfused placenta in the 1^st^ and 2^nd^ trimesters lead to excessive levels of circulating anti-endothelial factors. To elucidate the mechanism of GH or PE, and to develop a theory for GH or PE, appropriate animal models are effective.

Many animal models of GH or PE have been established according to various targets, which include immunological factors [16, 17]; abnormal placentation [18]; oxidative stress [19, 20]; and anti-endothelial factors [8, 9, 13], among others [21]. Models targeting anti-endothelial factors and oxidative stress are frequently used.

The GH or PE models were exploited. Some are rat and mouse models which were injected with adenoviral vector (AdV-) expressing sFLT-1 [8,9,22]. It resulted in increased mean arterial pressure, proteinuria, and glomerular endotheliosis. This model revealed high levels of serum sFLT-1 and GH or PE symptoms. However, the sFLT-1 expression in the rat model was mainly transient in the maternal liver, followed by intrahepatic hemorrhage or hepatotoxicity [23], then various agents are released from the liver. Therefore, the levels of endogenous concentration of sFLT-1 were unclear and were unsuitable for post-partum assessment.

To resolve this problem, we examined whether excessive placental sFLT-1 during pregnancy leads to GH or PE by creating mice models of HDP with specific placental overexpress of sFLT-1. The mice revealed hypertension and proteinuria at the concentrations of sFLT-1 that were comparable to the levels seen in human HDP. Additionally, fetal growth restriction was also observed, and both hypertension and proteinuria regressed after parturition.

Another GH or PE models was exploited from the point of oxidative stress wherein there was reduced uterine perfusion pressure (RUPP). This model mimics the physiological features of GH or PE in humans in terms of hypertension, proteinuria, and FGR. However, there are only a few studies of RUPP in mice [24, 25]. It is difficult to separate arteries from veins in mice, which are smaller than rats; on the other hand, sFLT-1 levels in the plasma did not differ between the sham and RUPP groups because of many miscarriage and resorption of embryos [25].

We established a GH mouse model by easy operative techniques and using the simple device. In the mouse model, we have enough time for observation of change from the time of operation to the onset of GH. We used ICR mice without genetic manipulations. Tightly narrowing aorta and vein led to an increase in the abortion **rate** and to decreased delivery of live pups according to the degree of narrowing them. We selected the degree of narrowing them, lest they did not influence the litter size. We narrowed both aorta and vein together using an inexpensive and readily available medical drip tube for appropriate level. The internal diameter of the tube was about 1.2 mm and the external diameter was about 2.2 mm. For ease of the procedure, we did not separate the artery and vein but we narrowed the artery and vein together. When we operated at day 10.5 of gestation, the abortion rate was lower and we could observe the mice for 8 days from operation to delivery, which was longer than in the RUPP rats that underwent operation at day 14.5 of gestation.

In the NAV mouse model, we recognized both increasing systolic and diastolic blood pressure at day 18.5 of gestation. In addition, we observed higher serum sFLT-1 level in the NAV group at day 18.5 of gestation compared with that in the control group, which is indeed characteristic of human GH.

NAV model was a GH model. Both serum sFLT-1 level and hypertension promptly normalized after delivery. This phenotype mimics human GH.

We established a simple GH mouse model through an easy operation using the simple device. This mouse model is suitable for developing treatments.

## Declarations

### Author contribution statement

Yuri Yasui: Performed the experiments; Contributed reagents, materials, analysis tools or data; Wrote the paper.

Keiichi Kumasawa: Conceived and designed the experiments; Performed the experiments; Analyzed and interpreted the data; Contributed reagents, materials, analysis tools or data; Wrote the paper.

Kennji Minato: Performed the experiments.

Hitomi Nakamura: Analyzed and interpreted the data.

Tadashi Kimura: Analyzed and interpreted the data.

### Funding statement

This work was supported by JSPS KAKENHI JP17K11232.

### Competing interest statement

The authors declare no conflict of interest.

### Additional information

No additional information is available for this paper.
